# Molecular and Clinical Characterization of a Founder Mutation Causing G6PC3 Deficiency

**DOI:** 10.1007/s10875-024-01836-0

**Published:** 2024-12-04

**Authors:** Xin Zhen, Michael J. Betti, Meltem Ece Kars, Andrew R. Patterson, Edgar Alejandro Medina-Torres, Selma Cecilia Scheffler Mendoza, Diana Andrea Herrera Sánchez, Gabriela Lopez-Herrera, Yevgeniya Svyryd, Osvaldo M. Mutchinick, Eric R. Gamazon, Jeffrey C. Rathmell, Yuval Itan, Janet Markle, Patricia O’Farrill Romanillos, Saul Oswaldo Lugo-Reyes, Ruben Martinez-Barricarte

**Affiliations:** 1 Division of Genetic Medicine, Department of Medicine, Vanderbilt University Medical Center, Nashville, TN USA; 2 Division of Molecular Pathogenesis, Department of Pathology Microbiology and Immunology, Vanderbilt University Medical Center, Nashville, TN USA; 3 Vanderbilt Genetics Institute, Vanderbilt University Medical Center, Nashville, TN USA; 4 The Charles Bronfman Institute for Personalized Medicine, Icahn School of Medicine at Mount Sinai, New York, NY USA; 5 Vanderbilt Center for Immunobiology, Nashville, TN USA; 6 Immune deficiencies laboratory, National Institute of Pediatrics, Health Secretariat, Mexico City, Mexico; 7 Clinical Immunology Service, National Institute of Pediatrics, Health Secretariat, Mexico City, Mexico; 8 Specialty Hospital, National Medical Center XXI Century, Mexico City, Mexico; 9 Department of Genetics, Instituto Nacional de Ciencias Médicas y Nutrición Salvador Zubirán, Mexico City, Mexico; 10 Vanderbilt Institute for Infection, Immunology and Inflammation, Nashville, TN USA; 11 Department of Genetics and Genomic Sciences, Icahn School of Medicine at Mount Sinai, New York, NY USA; 12 Mindich Child Health and Development Institute, Icahn School of Medicine at Mount Sinai, New York, NY USA

**Keywords:** Inborn errors of immunity, Primary immunodeficiency, Severe congenital neutropenia, G6PC3 deficiency, Founder effect, Metabolic dysfunction

## Abstract

G6PC3 deficiency is a monogenic immunometabolic disorder that causes severe congenital neutropenia type 4. Patients display heterogeneous extra-hematological manifestations, contributing to delayed diagnosis. Here, we investigated the origin and functional consequence of the *G6PC3* c.210delC variant found in patients of Mexican descent. Based on the shared haplotypes amongst mutation carriers, we estimated that this variant originated from a founder effect in a common ancestor. Furthermore, by ancestry analysis, we concluded that it appeared in the indigenous Mexican population. At the protein level, we showed that this frameshift mutation leads to an aberrant protein expression in overexpression and patient-derived Epstein-Barr Virus-immortalized B (EBV-B) cells. The neutropenia observed in G6PC3-deficient patients is driven by the intracellular accumulation of the metabolite 1,5-anhydroglucitol-6-phosphate (1,5-AG6P) that inhibits glycolysis. We characterized how the c.210delC variant impacts glycolysis by performing extracellular flux assays on patient-derived EBV-B cells. When treated with 1,5-anhydroglucitol (1,5-AG), the precursor to 1,5-AG6P, patient cells exhibited markedly reduced engagement of glycolysis. Finally, we compared the clinical presentation of patients with the mutation c.210delC and all other G6PC3-deficient patients reported in the literature, and we found that the c.210delC carriers display all prominent clinical features observed in prior patients. In conclusion, *G6PC3* c.210delC is a loss-of-function mutation that arose from a founder effect in the indigenous Mexican population. These findings may facilitate the diagnosis of additional patients in this geographical area. Moreover, the in vitro 1,5-AG-dependent functional assay used in our study could be employed to assess the pathogenicity of additional *G6PC3* variants.

**Supplementary Information:**

The online version contains supplementary material available at 10.1007/s10875-024-01836-0.

## Introduction

 Human glucose-6-phosphatase catalytic subunit 3 (G6PC3) deficiency is an autosomal recessive disorder first identified by Boztug et al. in 2009 as a cause of syndromic severe congenital neutropenia (SCN) [1]. In addition to accelerated apoptosis, neutrophils from patients show impaired chemotaxis and bactericidal activity [2, 3]. More than 110 patients have been described in the literature, and the clinical spectrum of G6PC3 deficiency continues to expand. Aside from neutropenia, the most frequently observed features include cardiac defects, urogenital malformations, prominent superficial veins, and endocrine abnormalities [4–6]. However, around 20% of the reported cases of G6PC3 deficiency manifest as a non-syndromic form of SCN [4, 7, 8]. Moreover, instances of cyclic neutropenia have been observed sporadically in patients [9, 10]. The phenotypic heterogeneity, coupled with the rarity of this disorder, contributes to delays in diagnosis and treatment.

In practice, it is often necessary to rule out several potential diagnoses before suspecting a genetic defect in patients with G6PC3 deficiency, and the process of identifying disease-causing variants through genetic testing afterward is intricate [11]. As most publicly available genetic databases were built using samples from individuals of European descent, alleles specific to other populations may not be well represented in these datasets [12, 13]. Thus, variants observed in patients from underrepresented ancestries are more prone to being classified as variants of uncertain significance (VUS), adding further complexity in achieving a prompt diagnosis [14]. Here, we established that the *G6PC3* c.210delC mutation, which was recurrently observed in patients from Mexico, originated from a founder effect and is of indigenous American ancestry. Furthermore, we described an effective functional assay to determine the deleteriousness of *G6PC3* variants using patient-derived Epstein-Barr Virus-immortalized B (EBV-B) cells.

## Materials and Methods

### Study Approval

This study was conducted in accordance with the Helsinki Declaration, with written informed consent obtained from the patients and their families. Approval for this study was obtained from the Vanderbilt University Medical Center Institutional Review Board (IRB), Nashville, USA.

### *G6PC3* Allele Age Estimation

Genomic DNA was isolated from whole blood samples using the GeneJET Whole Blood Genomic DNA Purification Kit (ThermoFisher Scientific, Waltham, MA). Primer sequences used to amplify exon 1 of *G6PC3* to confirm the c.210delC mutation through sanger sequencing were as follows: Forward: 5’- AGG AAA CGC CTT TAC AAA G -3’, Reverse: 5’- GAC AGT TGC TGA AAG AGA C -3’. Blood-derived DNA collected from 10 carriers of the *G6PC3* c.210delC allele (two trios and two mother-child pairs) were sequenced using Illumina short-read sequencing. The affected chromosome (chr17) was sequenced at an average read depth of 10.92x across all ten individuals. Raw reads were aligned to human reference genome build GRCh38/hg38 using Burrows-Wheeler Aligner [15] (0.7.17). PCR duplicates were subsequently identified in the aligned reads, and base quality scores were recalibrated following the Genome Analysis Toolkit (GATK) Best Practices Workflow [16]. Pre-called genomic variant call format (gVCF) from these six individuals underwent subsequent joint variant calling using the HaplotypeCaller [17] function from GATK (4.2.0.0). Finally, variant quality scores were recalibrated for small nucleotide polymorphisms (SNPs) and indels.

The jointly called variant call format (VCF) was pruned using VCFtools [18] (0.1.15) and VcfFilter from the BIOPET suite [19] (0.2). After removing variants with any missingness across the six individuals, those with a minor allele count of less than 3, a quality score of less than 30, or a minimum read depth of less than 20 were also removed.

Using this pruned variant set, the affected chromosome (chr17) was phased using SHAPEIT4 [20] (4.2.0), with 3,202 1000 Genomes samples sequenced at 30x [21] serving as the reference panel. The phased VCF was converted to hap/sample format using bcftools [22] (1.12). Using R (4.0.3), the hap file was reformatted as a 13-column text file containing the chr21 coordinate and phased variant calls for each of the 12 phased chromosomes (2 per individual) at that site. Phased variant calls could then be manually spot-checked for concordance with the aligned reads using the IGV genome browser [23] (2.9.4). As genetic data from the fathers were unavailable in two of the four pedigrees, these carrier haplotypes were inferred from the mother and proband haplotypes using a custom Python script. The lengths in centimorgans (cM) of the chr17 chromosomal arms upstream and downstream of the mutation site were calculated, and these respective lengths were used as inputs for the Mutation_age_estimation.R script developed by Gandolfo et al. [24]. A confidence coefficient of 0.95 without chance sharing correction was used, assuming a correlated genealogy.

### Ancestry Inference Using Principal Component Analysis (PCA)

To determine the genetic ancestries of the *G6PC3* c.210delC carriers, we combined the variant calls from 10 samples with those from 2,343 reference samples with African, American, and European ancestries from the harmonized HGDP + 1KGP dataset [25]. Variant filtering was performed according to Hardy-Weinberg equilibrium (*P* < 1 × 10^−6^) and missingness using PLINK v.1.9 [26]. Autosomal variants with a minor allele frequency > 1% were retained and pruned for linkage disequilibrium (*r*^2^ = 0.2). PCA was conducted using 157,601 variants with smartpca implemented in EIGENSOFT version 8.0.0 [27]. Eigenvectors were calculated using 2,343 reference samples, and the 10 *G6PC3* c.210delC carriers were projected onto these eigenvectors. A second PCA was performed using 8,032 variants located on chr17.

### Local Ancestry Estimation

Using the phase_common_static function from SHAPEIT (v5.5.1) [28], variant calls from the 10 sequenced carriers of the *G6PC3* c.210delC allele were phased together with 2,343 deeply sequenced reference samples (788 of European ancestry, 1,003 of African origin, and 552 of American ancestry) from the gnomAD v3.1.2 HGDP + 1KG callset [29] (https://gnomad.broadinstitute.org/downloads#v3-hgdp-1kg). After phasing, RFMix (v2.03-r0) [30] was used to estimate local ancestry across chr17 for the 10 carriers of the *G6PC3* c.210delC allele.

### Establishment of Epstein-Barr Virus-Immortalized B (EBV-B) Cell Lines

For generation of EBV-B cell lines derived from patients and healthy control individuals, B cells were immortalized with EBV as previously reported [31].

### Cell Culture

EBV-B cell lines were cultured in RPMI 1640 Medium without glucose (Gibco, Carlsbad, CA), supplemented with 5mM glucose (Gibco) and 10% fetal bovine serum (FBS) (Corning, Corning, NY). HEK293T cells (ATCC; CRL-3216) were cultured in DMEM medium (Sigma-Aldrich, St. Louis, MO) supplemented with 10% FBS (Corning).

### Plasmid Cloning and site-directed Mutagenesis

RNA was extracted from healthy control EBV-B cells using the RNeasy Plus Mini Kit (Qiagen, Germantown, MD). RNA was reverse transcribed using the Verso cDNA Synthesis Kit (ThermoFisher Scientific). Primer sequences used to generate the full-length cDNA of *G6PC3* with a His-tag right next to the start or stop codon of cDNA that encodes the G6PC3 protein were as follows: N-ter His-tag Forward: 5’- ATG CAT CAC CAT CAC CAT CAC ATG GAG TCC ACG CTG G -3’; N-ter His-tag Reverse: 5’- TCA GGA AGA GTG GAT GGG -3’; C-ter His-tag Forward: 5’- ATG GAG TCC ACG CTG G -3’; C-ter His-tag Reverse: 5’- TCA GTG ATG GTG ATG GTG ATG GGA AGA GTG GAT GGG C -3’. The products were TA-cloned into a pcDNA3.1 plasmid vector using the pcDNA 3.1/V5-His TOPO TA Expression Kit (ThermoFisher Scientific) according to the manufacturer’s protocol. Plasmid DNAs were purified from bacterial clones with a Miniprep kit (ThermoFisher Scientific). Then, constructs carrying the 210delC mutant allele were generated by site-directed mutagenesis with primers: Forward: 5’- CTC AAC CTC ATT TCA AGT GGT T -3’; Reverse: 5’- AAC CAC TTG AAA TGA GGT TGA G -3’. In brief, after the PCR reaction using PfuUltra II Fusion High-fidelity DNA Polymerase (Agilent, Santa Clara, CA), 1uL DpnI (NEB, Ipswich, MA) was added followed with incubation at 37 C for 3 hr. Mutagenesis was validated by Sanger sequencing.

### HEK293T Cell Transfection

In 6-well plates, HEK293T cells were transfected in 2 mL medium with 2.5ug of pcDNA3.1 empty vector, N or C-terminal His-tagged WT *G6PC3*, or N- or C-terminal His-tagged 210delC mutant constructs using Lipofectamine 3000 Transfection Reagent (ThermoFisher Scientific) according to the manufacturer’s protocol for 48 hr.

### *G6PC3* mRNA Detection by RT-qPCR

RNA was extracted from transfected HEK293T and EBV-B cells using the RNeasy Plus Mini Kit (Qiagen). The RT-qPCR was performed using the Luna Universal One-Step RT-qPCR Kit (NEB) on the CFX96 RT-qPCR detection system (Bio-Rad, Hercules, CA). The following primers were used to target the cDNA of *G6PC3*: Forward: 5’- TCA AGT GGT TTC TTT TTG GAG -3’; Reverse: 5’- ATC ATG CAG TGT CCA GAA G -3’. The *G6PC3* mRNA expression level in each sample was normalized to the expression level of the *GUS* gene transcript. Primers used to target *GUS* cDNA are: Forward: 5’- CTA CTT GAA GAT GGT GAT CG -3’; Reverse: 5’- CTG TTC AAA CAG ATC ACA TC -3’.

### Western Blotting Analysis

Whole-cell lysates were prepared from transfected HEK293T cells or EBV-B cells with RIPA lysis buffer (150 mM NaCl, 50 mM TRIS-HCl pH 8.0, 1 mM EDTA, 0.5% sodium deoxycholate, 1% NP-40, 0.1% SDS), supplemented with protease inhibitor cocktail (ThermoFisher Scientific). Protein concentrations were quantified with DC Protein Assay (Bio-Rad). To detect His tag, hexokinases, and ADP-GK, lysates mixed with Laemmli loading buffer were incubated at 100 °C for 5 min and subjected to electrophoresis in a 10% polyacrylamide gel. For analysis of LAMP2, lysates were mixed with XT sample buffer and reducing agent and subjected to electrophoresis in a precast 4–12% Bis-Tris Protein Gel using the MOPS running buffer (Bio-Rad). The membrane protein fraction of EBV-B cells was obtained with the Mem-PER Plus Membrane Protein Extraction Kit (ThermoFisher Scientific). The membrane protein extracts were mixed with loading buffer, incubated at 37 °C for 30 min, and subjected to electrophoresis in a precast 4–20% gradient gel (Bio-Rad). Proteins were then transferred to PVDF membranes, blocked with PBS-T containing 0.05% Tween-20 and 5% non-fat dry milk.

The following antibodies were used for western blotting: HRP-conjugated Anti-His tag (Biolegend, 652503, 1:5000), Anti-G6PC3 (Invitrogen, PA5-109749, 1:1000), Anti-ATP1A1 (Invitrogen, MA5-32184, 1:1000), HRP-conjugated Anti-GAPDH (Proteintech, HRP-60004, 1:1000), Anti-HK1 (Cell signaling, 2804, 1:1000), Anti-HK2 (Proteintech, 22029-1-AP, 1:3000), Anti-HK3 (Invitrogen, PA5-29304, 1:1000), Anti-ADPGK (Proteintech, 15639-1-AP, 1:1000), and Anti-LAMP2 (Santa Cruz Biotechnology, sc-18822, 1:300). Unconjugated antibodies were detected by incubation with HRP Goat Anti-mouse IgG secondary antibody (Sigma-Aldrich, AP127P, 1:5000) or HRP Goat Anti-Rabbit IgG secondary antibody (Sigma-Aldrich, AP156P, 1:5000). Blots were developed using the Pierce ECL Western Blotting Substrate (ThermoFisher Scientific).

### Extracellular Flux Assay

For EBV-B cells pretreatment prior to the glycolysis stress assay, cells were cultured in RPMI 1640 medium with 5mM glucose and 10% FBS, supplemented with either 2mM of 2-DG (Cayman Chemical, Ann Arbor, MI) or 1,5-AG (Cayman Chemical), for five days. Fresh medium was added to cells every two days. The assay was performed according to the Agilent Seahorse XF Glycolysis Stress Test Kit user guide. To summarize, EBV-B cells were counted and resuspended in Agilent Seahorse XF RPMI 1640 medium supplemented with 2mM glutamine, with 2mM of 2-DG or 1,5-AG added to corresponding conditioned cells. 150,000 live cells/well, in 180µL medium, were plated in 4–6 technical replicates on a Cell-Tak-coated plate (Corning). Extracellular acidification rate (ECAR) was measured on a Seahorse XFe 96 Analyzer using the glycolysis stress test with sequential injections of 20µL of 100mM glucose to Port A, 22µL of 15µM oligomycin to Port B, and 25µL of 500mM 2-DG to Port C of the sensor cartridge. The final concentrations of these compounds in each well are 10mM glucose, 1.5µM oligomycin, and 50mM 2-DG. The glycolysis rate was quantified as the change in ECAR before and after adding glucose. Glycolytic capacity was calculated as the difference between the maximal ECAR reached following the oligomycin injection and the non-glycolytic acidification prior to the addition of glucose.

### Viability Measurement of EBV-B cells

As described above, EBV-B cells were incubated with 2 mM of 2-DG or 1,5-AG for five days. Cells were stained with Zombie NIR dye (Biolegend) at room temperature for 15 min. Data was acquired using a BD LSRFortessa III flow cytometer (BD Biosciences). Using the FlowJo software v.10.10.0, the percentage of viable cells was quantified after gating on the singlet population using forward scatter (FSC) and side scatter (SSC).

## Results

### The *G6PC3* c.210delC Variant Found in Mexico Originated from a Founder Effect

**Fig. 1:**
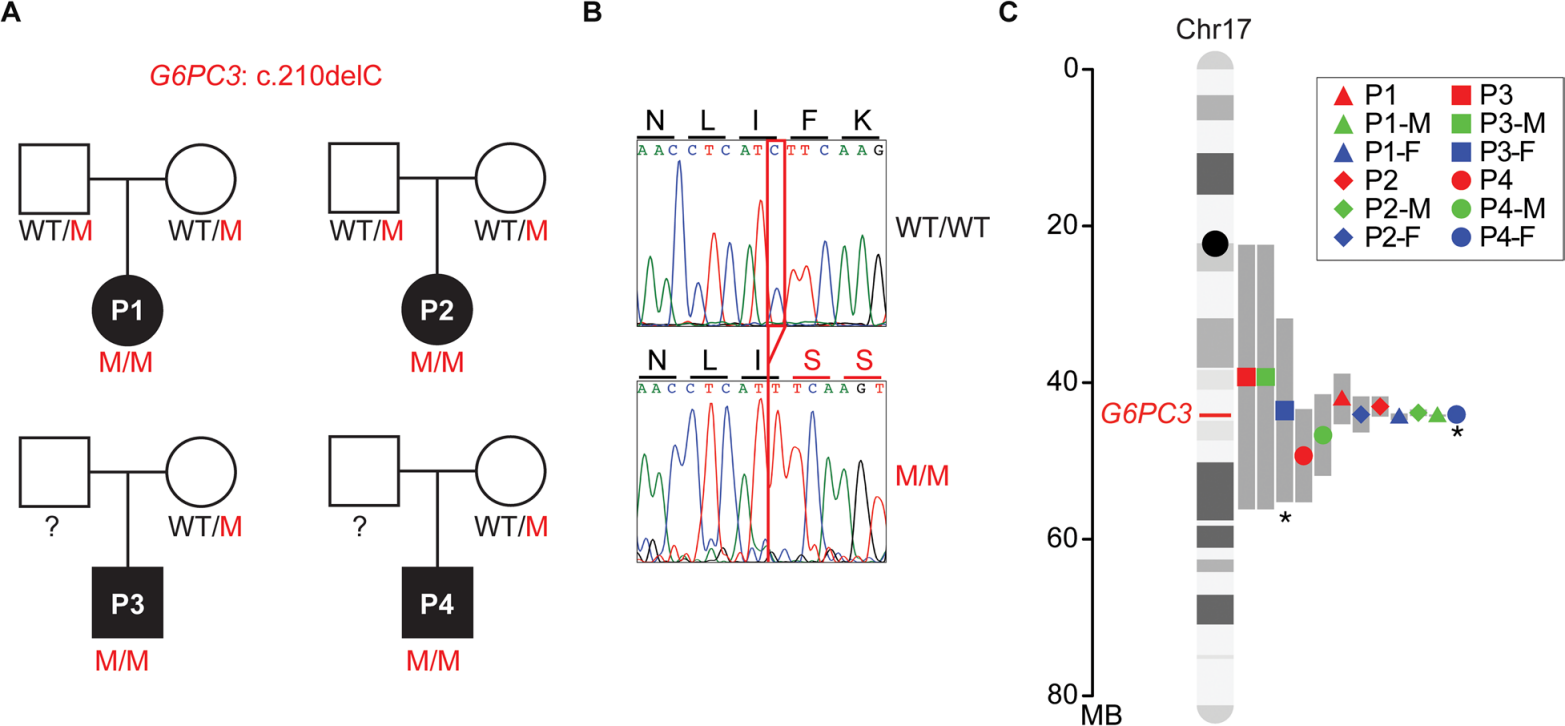
A common ancestor in patients with the *G6PC3*c.210delC mutation.(**A**) Familial segregation of the mutation in four unrelated, non-consanguineous families. WT: wild type; M: mutant. **(B)** Sanger sequencing results of a healthy control and patient 1 (P1) in the region spanning the *G6PC3* c.210delC mutation. Amino acid changes resulting from the mutation are annotated above the graphs. **(C)** Age estimation for the *G6PC3* c.210delC variant, based on the lengths of shared ancestral haplotype blocks upstream and downstream of the G6PC3 frameshift allele (marked in red on chr17). The computationally inferred haplotypes from Father 3 (P3-F) and Father 4 (P4-F) are denoted with an asterisk

Among all the mutations causing G6PC3 deficiency, a few are only found in specific ethnic groups, implying founder effects [6]. For instance, the c.210delC variant has been reported in 13 G6PC3-deficient patients, who are either homozygous or compound heterozygous for this variant [4, 32–34]. Among them, 12 patients are of Mexican descent, while another patient is included in the North American Severe Chronic Neutropenia International Registry [32]. These observations led us to interrogate whether a founder effect causes the recurrence of this mutation, or if it is a mutational hotspot. To study this, we recruited four patients from central Mexico who are homozygous for this variant and were born to unrelated, non-consanguineous parents (Fig. 1A-B). We collected genomic DNA from these four patients plus six of their heterozygous healthy parents. We performed whole-genome sequencing (WGS) followed by haplotype analysis on the region of chromosome 17 surrounding *G6PC3*. Since samples were unavailable from two of the patients’ fathers, these two carrier haplotypes were inferred computationally from the mother and patient haplotypes. Our analysis revealed a shared haplotype segment surrounding the mutation site among all carriers (Fig. 1C). The length of this shared haplotype suggests that the mutation originated 26 generations ago (95% confidence interval, 2.3–50.5) in a common ancestor. This analysis indicates that the *G6PC3* c.210delC is a single-nucleotide deletion originated through a founder effect.

### The *G6PC3* c.210delC Mutation is of Indigenous American Origin

**Fig. 2:**
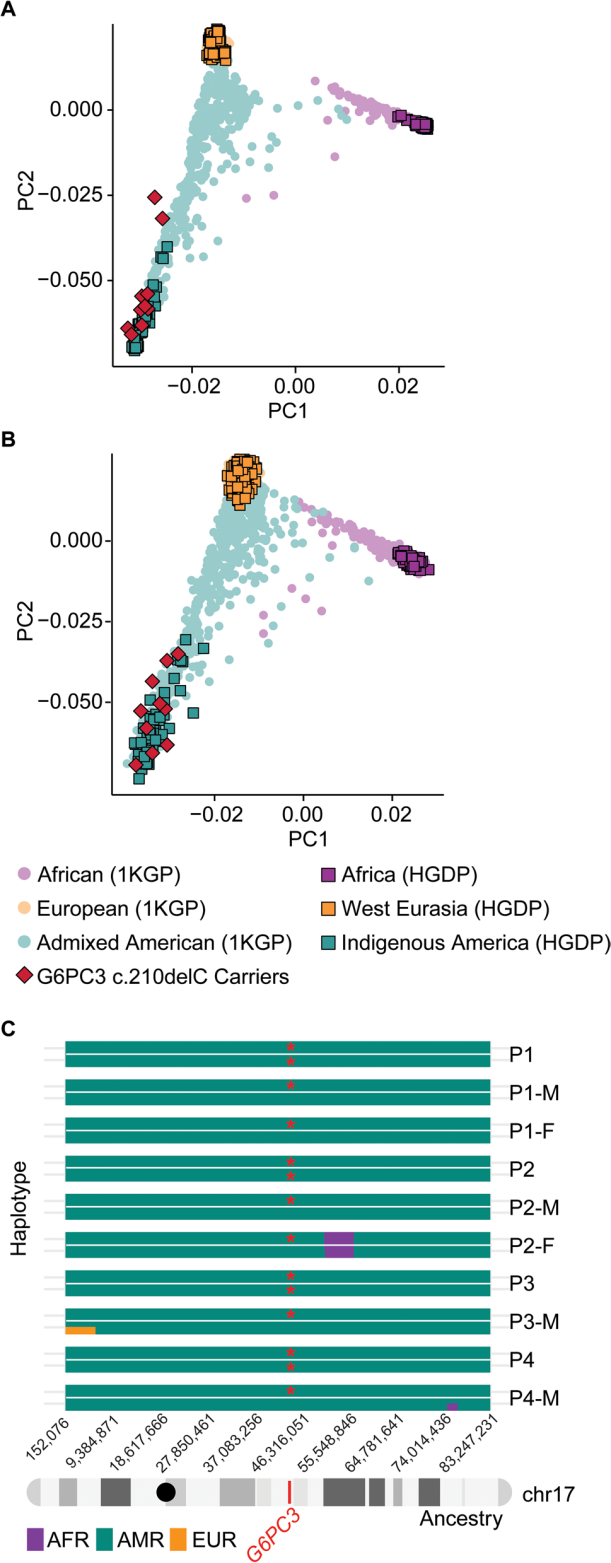
The *G6PC3* c.210delC mutation is estimated to be of indigenous American ancestry. Principal component analysis (PCA) of ancestry on (**A**) the whole genome for c.210delC mutation carriers, using reference genomes from the combined 1000 Genomes Project (1kGP) and Human Genome Diversity Project (HGDP) dataset, and **(B)** on chromosome 17. **(C)** Local ancestry estimation across carriers of the c.210delC variant. The mutation locus (chr17:44,071,175) is marked in red on chromosome 17. Mutated alleles are labeled with red asterisks. AFR: African ancestry; AMR: Admixed American ancestry; EUR: European ancestry

Based on the assumption that each generation interval is between 20 and 25 years, the estimated age of the *G6PC3* c.210delC variant is 520–650 years. This allele age raised the question of whether the shared haplotype originated from native American ancestry or was introduced to Mexico by Europeans. To investigate this, we conducted principal component analysis (PCA) with WGS data of the 10 carriers, using reference populations from the combined 1000 Genomes Project (1KGP) and Human Genome Diversity Project (HGDP) dataset. Ancestry inference analysis on the whole genome and chromosome 17 showed that the carriers of this mutation cluster closely with the indigenous American populations from HGDP (Fig. 2A-B). Moreover, we used 2,343 deeply sequenced reference samples of European, African, and Ad Mixed American Ancestry individuals from the 1KGP + HGDP dataset to perform local ancestry estimation across chromosome 17. Our analysis indicates that the chromosomal region containing the *G6PC3* c.210delC mutation (chr17: 44,071,175) is of American origin (Fig. 2C). Overall, these findings suggest that the mutation c.210delC originated in the indigenous Mexican population.

### The *G6PC3* c.210delC Variant Results in Reduced *G6PC3* mRNA and Complete Loss of Protein Expression

**Fig. 3:**
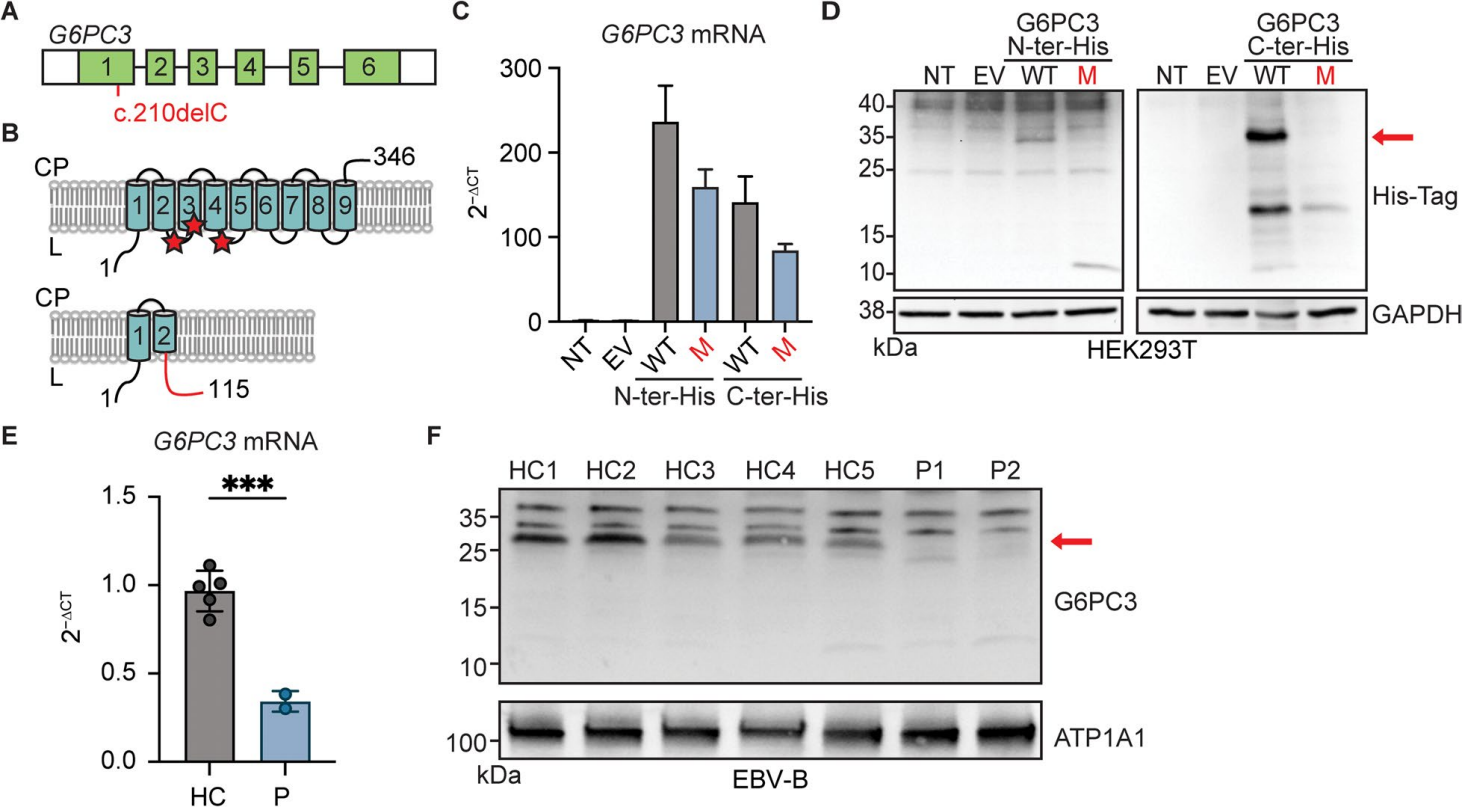
The *G6PC3* c.210delC mutation leads to a complete loss of protein expression. Schematic representation of (**A**)the *G6PC3* gene with each box representing an exon and the mutation indicated in red, and **(B)** the G6PC3 protein structure with nine transmembrane domains in the endoplasmic reticulum. Red stars indicate the active site. The lower panel shows the predicted consequence of the c.210delC (p.F71Sfs*46) mutation. The out-of-frame sequence resulting from the premature stop codon is indicated in red. CP: cytoplasm; L: lumen. **(C)** RT-qPCR of *G6PC3* mRNA expression in HEK293T cells either non-transfected (NT), transfected with the empty vector (EV), or transfected with plasmids encoding wildtype (WT) or F71Sfs*46 (M) G6PC3 with a 6xHis tag at either the N- or C-terminal. *GUS* was used as a control for gene expression. *n* = 3. **(D)** Western blotting of transfected HEK293T cell lysates using an anti-His Tag antibody. GAPDH was used as a loading control. **(E)** RT-qPCR of *G6PC3* mRNA expression in EBV-B cells from patients and healthy controls. Data are presented as mean ± SD and represent three independent experiments. ***p ≤ 0.001 in a Student’s t-test. **(F)** G6PC3 protein expression by western blotting in membrane protein fraction of EBV-B cells. ATP1A1 was used as a membrane protein loading control

*G6PC3* encodes a protein with nine transmembrane domains that localizes to the endoplasmic reticulum [35, 36]. The c.210delC variant observed in our patients is a single-nucleotide deletion in exon 1 of *G6PC3* (Fig. 3A). It is predicted to cause a shift in the reading frame after amino acid 70 (in the second transmembrane domain of the protein), thus introducing a premature stop codon at 46 amino acids after the mutation site (p.F71Sfs*46) (Fig. 3B). To examine the impact of this variant at mRNA and protein levels, we first transfected human embryonic kidney 293T (HEK293T) cells with plasmids containing C- or N-terminally polyhistidine-tagged versions of wildtype (WT) and p.F71Sfs*46 G6PC3. RT-qPCR results suggested that the mutation induces a moderate reduction in *G6PC3* mRNA levels (Fig. 3C). Western blotting of whole cell lysates with an anti-His-tag antibody yielded a 10 kDa band in the cells transfected with N-terminal tagged p.F71Sfs*46 G6PC3, aligning with the anticipated molecular weight of a truncated mutant protein (Fig. 3D). This predicted mutant protein would not retain enzymatic activity as it lacks the active site of G6PC3, which is composed of amino acids R79, H114, and H167 [35, 36] (Fig. 3B). No band was observed by western blotting using lysate of cells transfected with the C-terminal His-tagged mutant G6PC3 (Fig. 3D). This validates the absence of a translation reinitiation downstream of the premature termination codon, a mechanism that can yield partially functional proteins in the context of mutations causing other inborn errors of immunity [37–39]. We utilized Epstein-Barr Virus immortalized B (EBV-B) cells derived from two patients and healthy controls to gain additional insights in a more physiologically relevant context. We observed markedly reduced levels of *G6PC3* mRNA in patient cells, potentially due to nonsense-mediated mRNA decay (Fig. 3E). Furthermore, using membrane protein fractions extracted from EBV-B cells for western blotting and a polyclonal antibody with epitope spanning the N-terminal region of human G6PC3, we showed that neither the WT nor the mutant size protein was expressed in cells from the patients (Fig. 3F). Altogether, these results demonstrate that the *G6PC3* c.210delC (p.F71Sfs*46) variant disrupts mRNA and protein levels, causing a complete loss of expression.

### Cells from Patients with the *G6PC3* c.210delC Variant Show Normal Viability and Glycosylation Pattern but Impaired Glycolysis

G6PC3 is a metabolite-repair enzyme involved in the hydrolysis of 1,5-anhydroglucitol-6-phosphate (1,5-AG6P), which is the phosphorylated form of a common food-derived polyol named 1,5-anhydroglucitol (1,5-AG) [40]. 1,5-AG is normally present in blood and is considered metabolically inert, since the balance between oral intake and urinary excretion of 1,5-AG maintains a body pool of about 500-1,000 mg in normoglycemia [41, 42]. After entering the cytoplasm, 1,5-AG can be phosphorylated into 1,5-AG6P by side reactions of low-K_M_ hexokinases and ADP-glucokinase (ADP-GK). Then, 1,5-AG6P enters the endoplasmic reticulum and can be dephosphorylated by G6PC3 [40]. When a functional G6PC3 is absent, 1,5-AG6P can accumulate to a concentration that inhibits hexokinase activity [40]. Since hexokinase mediates the rate-limiting first step of the glycolytic pathway, intracellular accumulation of 1,5-AG6P impairs glycolysis [43]. This mechanism prompts cell death in G6PC3-deficient neutrophils since neutrophils rely heavily on glycolysis to fulfill their energetic needs [40,44].

**Fig. 4:**
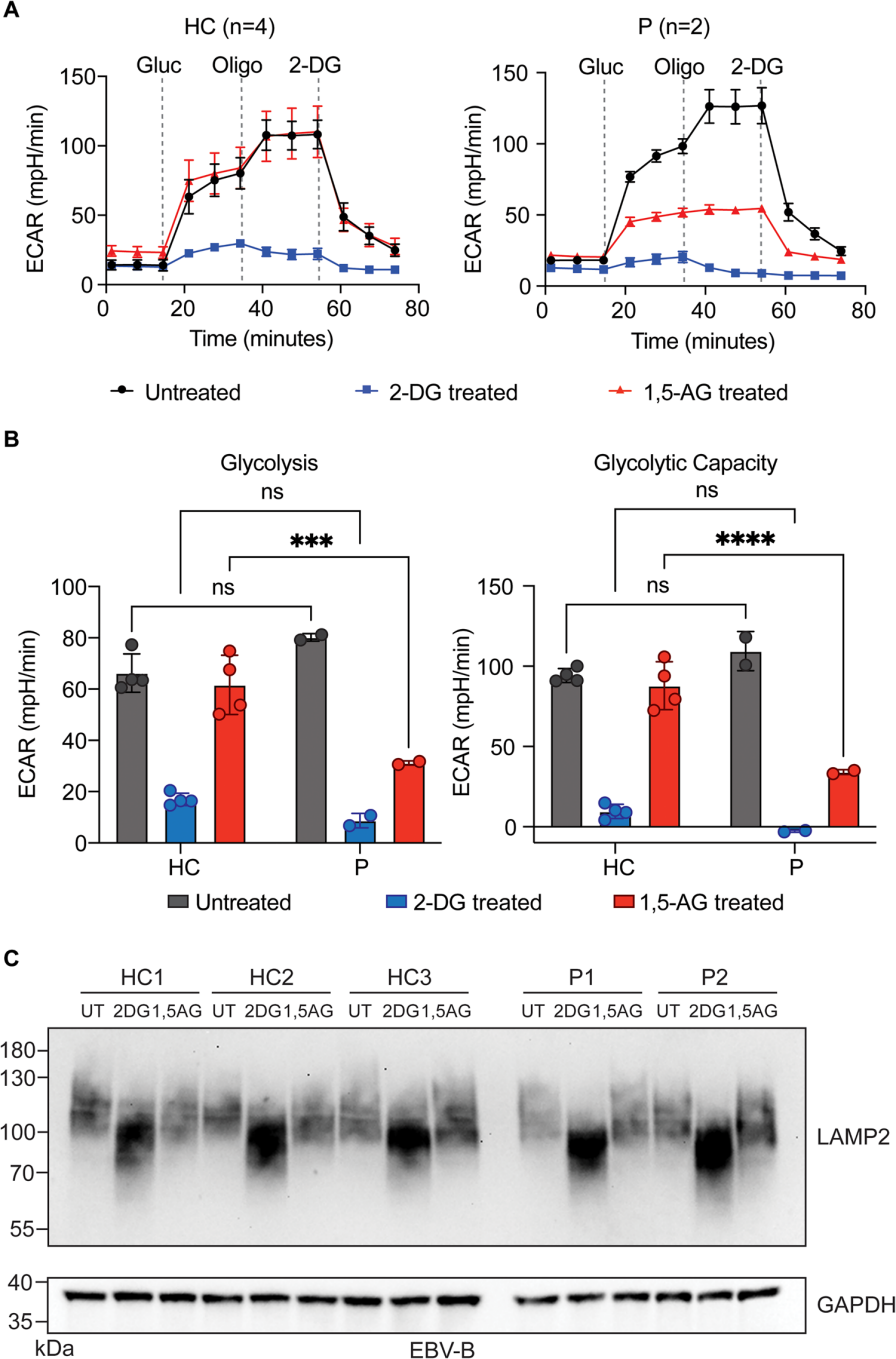
Impaired glycolysis in cells from G6PC3-deficient patients.(**A**) Measurement of extracellular acidification rate (ECAR) in response to glucose (gluc), ATP synthase inhibitor oligomycin (oligo), and glycolytic inhibitor 2-deoxy-glucose (2-DG) in EBV-B cells from two patients (P) and four healthy controls (HC) pretreated with 2-DG or 1,5-AG. **(B)** Quantification of glycolysis rate and glycolytic capacity in EBV-B cells from four healthy controls and two patients. **(C)** Western blotting analysis of LAMP2 glycosylation pattern in EBV-B cells. Cells were left untreated (UT), or treated with 2-DG or 1,5-AG for five days before the preparation of whole cell lysates. GAPDH was used as a loading control. Data are presented as mean ± SD and represent three independent experiments. Statistical analysis was performed using two-way ANOVA with Šidák correction. ***p ≤ 0.001, ****p ≤ 0.0001

To directly assess the consequence of the absence of G6PC3 caused by the c.210delC variant, we used patient-derived EBV-B cells to determine if they have defective hexokinase activity. EBV-B cells express all isoforms of low-K_M_ hexokinases (HK1, HK2, and HK3) and ADP-GK identified in mammalian cells, thus permitting us to test the functional impact of the G6PC3 deficiency (Fig. S1). EBV-B cells derived from patients and healthy controls were treated with either 1,5-AG or 2-Deoxy-D-Glucose (2-DG) for five days. 2-DG, a widely used potent inhibitor of hexokinases and, hence, of glucose metabolism, was utilized as a positive control for glycolytic inhibition [45]. Considering the toxicity of 1,5-AG6P accumulation in G6PC3-deficient neutrophils, we validated that the 5-day 1,5-AG treatment has no impact on the viability of EBV-B cells [40] (Fig. S2). This finding aligns with the immunological features of G6PC3-deficient patients, where neutropenia emerges as the most pronounced hematological abnormality, suggesting that the effect of 1,5-AG6P accumulation on other immune cells is much subtler, possibly attributable to their metabolic plasticity. We then quantified the glycolytic activities of our untreated, 1,5-AG-treated, and 2-DG-treated EBV-B cells through extracellular acidification rate (ECAR) measurements. Patient EBV-B cells exhibited similar levels of glycolytic activity to cells derived from healthy controls, whether untreated or treated with 2-DG. However, patient cells showed significantly impaired glycolysis rate and glycolytic capacity following the 1,5-AG treatment, while healthy control cells remained unaffected (Fig. 4A-B). These results illustrate the metabolic disturbance in cells from patients with the *G6PC3* c.210delC variant due to the absence of G6PC3, establishing ECAR measurements in EBV-B cells as a means to effectively evaluate the functional consequences of *G6PC3* mutations. Apart from glycolytic impairment, suppression of hexokinase activity can also lead to abnormal protein glycosylation, manifested as decreased glycosylation of the heavily glycosylated protein LAMP2 in patient neutrophils [46–48]. We assessed the glycosylation state of LAMP2 in EBV-B cells from patients and healthy controls by western blotting. 1,5-AG-treated cells showed no defect in LAMP2 glycosylation. At the same time, a smear with lower molecular weight was observed in cells treated with 2-DG, indicating hypo-glycosylation of LAMP2 (Fig. 4C). These data suggest that glycolysis is the most significantly affected pathway in patient-derived EBV-B cells that can reflect the deleteriousness of a *G6PC3* mutation.

### Patients with the *G6PC3* c.210delC Variant Show a Clinical Profile Similar to Other G6PC3-Deficient Patients

**Fig. 5:**
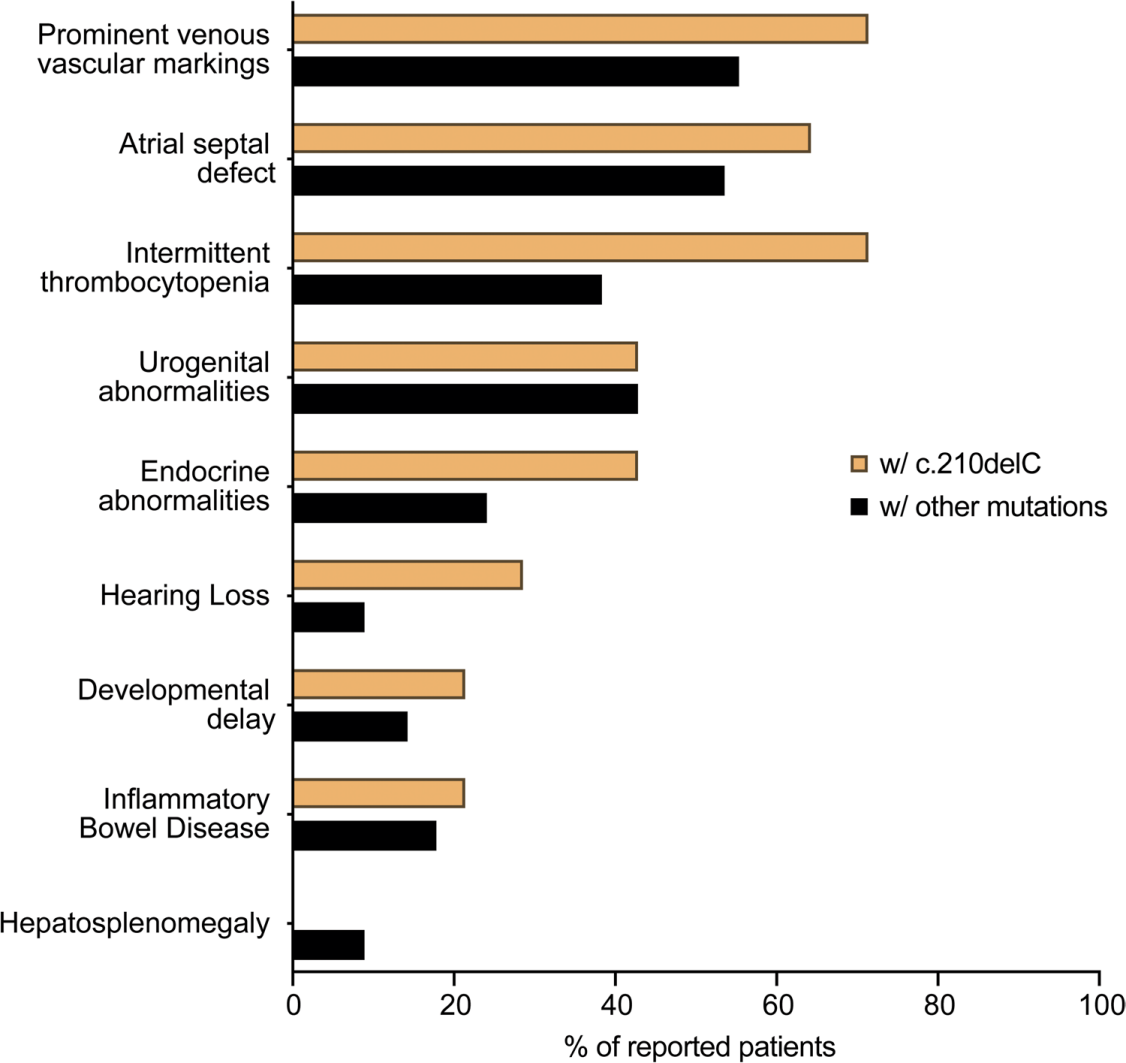
Frequencies of the most prominent clinical features observed in G6PC3-deficient patients with the c.210delC mutation (*n* = 14) and those with other mutations (*n* = 112)

As all patients with the *G6PC3* c.210delC variant are from the same geographical area, we evaluated whether they exhibit any characteristic in their clinical presentation that may differentiate them from the rest of the reported G6PC3-deficient patients, denoting some environmental aspects of the disease. To this end, we collected the clinical information from all published cases of G6PC3 deficiency to compare the frequency of appearance of nine prominent clinical features between patients without and with the *G6PC3* c.210delC mutation (*n* = 14, some published, some unpublished) (Fig. 5). None of the patients with the *G6PC3* c.210delC mutation showed isolated neutropenia but presented with features of syndromic severe congenital neutropenia including extra-hematological abnormalities. Except for hepatosplenomegaly, all other features of G6PC3 deficiency have been observed in these patients. We also noticed that patients with the c.210delC variant display a higher occurrence of thrombocytopenia, endocrine abnormalities, and hearing loss. Although this may imply a specific characteristic of this group of patients, it might also reflect some variability in standard clinical testing. Overall, our analysis shows that patients who are carriers of the *G6PC3* c.210delC mutation display all main clinical characteristics described in G6PC3 deficiency, indicating that the mutation may be the main driver of their disease.

## Discussion

G6PC3 deficiency is a rare genetic disorder with a broad phenotypic spectrum, posing difficulties for timely diagnosis. The differential diagnosis process can be particularly complicated for patients with non-syndromic neutropenia or less frequently observed clinical features [49–51]. Mutations observed in G6PC3-deficient patients spread across all six exons of the gene [6]. Interestingly, the prevalence of several G6PC3 alleles varies significantly amongst different ethnic groups. These include the p.Pro44Ser mutation of Pakistani origin and the p.W73X mutation in patients from the Dominican Republic [52, 53]. In this study, through haplotype analysis and ancestry inference, we demonstrated that the *G6PC3* c.210delC variant was recurrently observed in Mexico due to a founder effect, and it is of native American origin. We also reviewed the signs and symptoms in patients with this variant, concluding that they closely resemble those observed in other reported patients. These findings may facilitate targeted testing of patients from this region with unexplained congenital neutropenia.

The mechanisms responsible for the phenotypic variability in G6CP3 deficiency remain elusive thus far [6, 8, 54]. It has been hypothesized that these variations might be correlated with the residual G6PC3 enzymatic activity resulting from some mutations [55]. To assess the functional impact of mutations, early studies measured the capacity of mutant G6PC3 in mediating hydrolysis of glucose-6-phosphate (G6P) using microsomes isolated from transfected yeast cells or COS-1 cell homogenates [1, 55]. However, it has been recently established that the primary physiological role of G6PC3 is not to dephosphorylate G6P into glucose and phosphate. Instead, the molecular mechanism underlying neutrophil dysfunction observed in G6PC3-deficient patients is associated with inhibited hexokinase activity and accumulation of 1,5-AG6P [40, 47]. In this regard, several methods have been used to study the functional consequence of *G6PC3* mutations. One approach is to directly quantify the concentration of 1,5-AG6P in neutrophils obtained from patients by liquid chromatography-mass spectrometry (LC-MS) [40, 47]. Alternatively, one could generate recombinant wildtype and mutant G6PC3 proteins and compare their phosphatase activity in dephosphorylating 1,5-AG6P [40]. A major drawback inherent to these approaches is a lack of commercial availability of both 1,5-AG6P, which is used as the substrate in the phosphatase assay, and its deuterated form, employed as the internal standard in LC-MS analysis. Thus, these compounds need to be synthesized chemically from the precursor of 1,5-AG. In addition, analysis of the glycosylation state of LAMP2 in patient neutrophils holds the potential to reflect the enzymatic activity of G6PC3 [46–48]. Nevertheless, it is worth noting that isolating a sufficient number of neutrophils from blood samples of neutropenic patients can be challenging, particularly when samples need to be transported for testing. Furthermore, glycosylation may be altered in immune cells by factors independent of hexokinase inhibition, including genetic and environmental cues [56].

Here, we demonstrated that patient-derived EBV-B cells can be utilized to assess the pathogenicity of *G6PC3* variants effectively. Compared to primary neutrophils, EBV-B cell lines can be generated from a small amount of patient blood samples and undergo unlimited proliferation in culture. The generation of EBV-B cells also preserves the genetic information of each patient, and they allow measurement of endogenous levels of G6PC3 [57]. Results of our extracellular flux assay show that patient EBV-B cells, when treated with 1,5-AG, exhibited defective glycolytic activities, indicating that the *G6PC3* c.210delC variant disrupts the metabolite repair activity of G6PC3 in eliminating 1,5-AG6P. Furthermore, this system allows one to perform genetic rescue experiments to validate that a specific variant in *G6PC3* causes impaired function. This in vitro assay could be employed in future studies to quantify the impact of *G6PC3* mutations in additional patients, which would aid in establishing potential genotype-phenotype correlations for this disease and predicting the pathogenicity of VUS.

In conclusion, this study identified the *G6PC3* c.210delC allele as a founder mutation that abolishes protein expression and function. These findings may help expedite the diagnosis of G6PC3 deficiency, especially in the Mexican population. As previous reports suggested that G6PC3 deficiency can lead to death from severe infections when neutropenia is left untreated, prompt diagnosis and provision of treatments are critical [58, 59]. Importantly, empagliflozin, a sodium-glucose cotransporter 2 (SGLT2) inhibitor frequently used to treat type 2 diabetes, has successfully resolved neutrophil defects in patients with G6PC3 deficiency by lowering the 1,5-AG blood concentrations [47, 60, 61]. Along with using this highly effective, safe, and easy-to-take oral alternative to granulocyte-colony-stimulating factor (G-CSF) injections, early disease diagnosis may improve outcomes of G6PC3-deficient patients [48, 62].

## Supplementary Information

Below is the link to the supplementary material.

ESM 1 (DOCX 2.39 MB)

## Data Availability

The data supporting the findings of this study are provided within the manuscript and supplementary materials, or are available upon reasonable request from the corresponding authors.

## References

[1] Boztug K, et al. A syndrome with congenital neutropenia and mutations in G6PC3. N Engl J Med. 2009;360:32–43. 19118303 10.1056/NEJMoa0805051 PMC2778311

[2] McKinney C, et al. Metabolic abnormalities in G6PC3-deficient human neutrophils result in severe functional defects. Blood Adv. 2020;4:5888. 33259599 10.1182/bloodadvances.2020002225 PMC7724913

[3] Dai R, et al. Altered functions of neutrophils in two Chinese patients with severe congenital neutropenia type 4 caused by G6PC3 mutations. Front Immunol. 2021;12:699743. 34305938 10.3389/fimmu.2021.699743 PMC8296982

[4] Velez-Tirado N, et al. Severe congenital neutropenia due to G6PC3 deficiency: case series of five patients and literature review. Scand J Immunol. 2022;95:e13136. 34964150 10.1111/sji.13136

[5] Maroufi SF, et al. Novel G6PC3 mutations in patients with congenital neutropenia: case reports and review of the literature. Endocr Metab Immune Disord Drug Targets. 2021;21:1660–8. 34137364 10.2174/1871530321666210616110631

[6] Banka S, Newman WG. A clinical and molecular review of ubiquitous glucose-6-phosphatase deficiency caused by G6PC3 mutations. Orphanet J Rare Dis. 2013;8:84. 23758768 10.1186/1750-1172-8-84 PMC3718741

[7] Khera S, Pramanik SK, Patnaik SK. A novel mutation in G6PC3 gene associated non-syndromic severe congenital neutropenia. Indian Pediatr. 2020;57:574–5. 32562405

[8] Banka S, Wynn R, Byers H, Arkwright PD, Newman WG. G6PC3 mutations cause non-syndromic severe congenital neutropenia. Mol Genet Metab. 2013;108:138–41. 23298686 10.1016/j.ymgme.2012.12.001

[9] Bruni CM, de la Rua W, Sadre SY, Nestor JM, Ahmed R. A novel pathogenic variant of G6PC3 gene presenting as cyclic Neutropenia in a pediatric patient. Blood. 2021;138:4191.

[10] Alangari AA, Alsultan A, Osman ME, Anazi S, Alkuraya F. A novel homozygous mutation in G6PC3 presenting as cyclic neutropenia and severe congenital neutropenia in the same family. J Clin Immunol. 2013;33:1403–6. 24105461 10.1007/s10875-013-9945-7

[11] Kiykim A, et al. G6PC3 deficiency: primary immune deficiency beyond just neutropenia. J Pediatr Hematol Oncol. 2015;37:616–22. 26479985 10.1097/MPH.0000000000000441

[12] Gudmundsson S, et al. Variant interpretation using population databases: lessons from gnomAD. Hum Mutat. 2022;43:1012. 34859531 10.1002/humu.24309 PMC9160216

[13] Sirugo G, Williams SM, Tishkoff SA. The missing diversity in human genetic studies. Cell. 2019;177:26. 30901543 10.1016/j.cell.2019.02.048 PMC7380073

[14] Chen E, et al. Rates and classification of variants of uncertain significance in hereditary disease genetic testing. JAMA Netw Open. 2023;6:E2339571. 37878314 10.1001/jamanetworkopen.2023.39571 PMC10600581

[15] Li H, Durbin R. Fast and accurate short read alignment with Burrows-Wheeler transform. Bioinformatics. 2009;25:1754–60. 19451168 10.1093/bioinformatics/btp324 PMC2705234

[16] McKenna A, et al. The genome analysis toolkit: a MapReduce framework for analyzing next-generation DNA sequencing data. Genome Res. 2010;20:1297–303. 20644199 10.1101/gr.107524.110 PMC2928508

[17] Poplin R, et al. Scaling accurate genetic variant discovery to tens of thousands of samples. bioRxiv. 2018;201178. 10.1101/201178.

[18] Danecek P, et al. The variant call format and VCFtools. Bioinformatics. 2011;27:2156. 21653522 10.1093/bioinformatics/btr330 PMC3137218

[19] Hof, P. V. T., et al. BIOPET: towards scalable, maintainable, user-friendly, robust and flexible NGS data analysis pipelines. Proceedings − 2017 17th IEEE/ACM International Symposium on Cluster, Cloud and Grid Computing, CCGRID 2017. 2017. pp. 823–829. 10.1109/CCGRID.2017.59.

[20] Delaneau O, Zagury JF, Robinson MR, Marchini JL, Dermitzakis ET. Accurate, scalable and integrative haplotype estimation. Nature Communications. 2019;10:1–10. 10.1038/s41467-019-13225-y PMC6882857 31780650

[21] Byrska-Bishop M, et al. High-coverage whole-genome sequencing of the expanded 1000 genomes project cohort including 602 trios. Cell. 2022;185:3426–e344019. 36055201 10.1016/j.cell.2022.08.004 PMC9439720

[22] Danecek P, et al. Twelve years of SAMtools and BCFtools. Gigascience. 2021;10:1–4. 10.1093/gigascience/giab008 PMC7931819 33590861

[23] Thorvaldsdóttir H, Robinson JT, Mesirov JP. Integrative Genomics Viewer (IGV): high-performance genomics data visualization and exploration. Brief Bioinform. 2013;14:178–92. 22517427 10.1093/bib/bbs017 PMC3603213

[24] Gandolfo LC, Bahlo M, Speed TP. Dating rare mutations from small samples with dense marker data. Genetics. 2014;197:1315–27. 24879464 10.1534/genetics.114.164616 PMC4125402

[25] Koenig Z, et al. A harmonized public resource of deeply sequenced diverse human genomes. bioRxiv. 2024;20230123525248. 10.1101/2023.01.23.525248. 10.1101/gr.278378.123 PMC11216312 38749656

[26] Purcell S, et al. PLINK: a tool set for whole-genome association and population-based linkage analyses. Am J Hum Genet. 2007;81:559–75. 17701901 10.1086/519795 PMC1950838

[27] Price AL, et al. Principal components analysis corrects for stratification in genome-wide association studies. Nat Genet. 2006;38(8):904–9. 16862161 10.1038/ng1847

[28] Hofmeister RJ, Ribeiro DM, Rubinacci S, Delaneau O. Accurate rare variant phasing of whole-genome and whole-exome sequencing data in the UK Biobank. Nat Genet. 2023;55(7):1243–9. 37386248 10.1038/s41588-023-01415-w PMC10335929

[29] Koenig Z, et al. A harmonized public resource of deeply sequenced diverse human genomes. bioRxiv. 2023;20230123525248. 10.1101/2023.01.23.525248. 10.1101/gr.278378.123 PMC11216312 38749656

[30] Maples BK, Gravel S, Kenny EE, Bustamante CD. RFMix: a discriminative modeling approach for rapid and robust local-ancestry inference. Am J Hum Genet. 2013;93:278. 23910464 10.1016/j.ajhg.2013.06.020 PMC3738819

[31] Tosato G, Cohen JI. Generation of Epstein-Barr Virus (EBV)–immortalized B cell lines. Curr Protoc Immunol. 2007;76:7.22.1-7.22.4. 10.1002/0471142735.im0722s76 18432996

[32] Xia J, et al. Prevalence of mutations in ELANE, GFI1, HAX1, SBDS, WAS and G6PC3 in patients with severe congenital neutropenia. Br J Haematol. 2009;147:535–42. 19775295 10.1111/j.1365-2141.2009.07888.x PMC2783282

[33] Boztug K, et al. Extended spectrum of human glucose-6-phosphatase catalytic subunit 3 deficiency: novel genotypes and phenotypic variability in severe congenital neutropenia. J Pediatr. 2012;160(4):679–83. 22050868 10.1016/j.jpeds.2011.09.019

[34] López-Rodríguez L, et al. Severe congenital neutropenia type 4: a rare disease harboring a G6pc3 gene pathogenic variant particular to the mexican population. Rev Invest Clin. 2022;74:328–39. 36546889 10.24875/RIC.22000234

[35] Guionie O, Clottes E, Stafford K, Burchell A. Identification and characterisation of a new human glucose-6-phosphatase isoform. FEBS Lett. 2003;551:159–64. 12965222 10.1016/s0014-5793(03)00903-7

[36] Shieh JJ, Pan CJ, Mansfield BC, Chou JY. A glucose-6-phosphate hydrolase, widely expressed outside the liver, can explain age-dependent resolution of hypoglycemia in glycogen storage disease type Ia. J Biol Chem. 2003;278:47098–103. 13129915 10.1074/jbc.M309472200

[37] Moey C, et al. Reinitiation of mRNA translation in a patient with X-linked infantile spasms with a protein-truncating variant in ARX. Eur J Hum Genet. 2016;24:681–9. 26306640 10.1038/ejhg.2015.176 PMC4930085

[38] Puel A, et al. The NEMO mutation creating the most-upstream premature stop codon is hypomorphic because of a reinitiation of translation. Am J Hum Genet. 2006;78:691–701. 16532398 10.1086/501532 PMC1424680

[39] Asano T, et al. Human STAT3 variants underlie autosomal dominant hyper-IgE syndrome by negative dominance. J Exp Med. 2021;218(8): e20202592. 10.1084/jem.20202592 PMC8217968 34137790

[40] Veiga-da-Cunha M, et al. Failure to eliminate a phosphorylated glucose analog leads to neutropenia in patients with G6PT and G6PC3 deficiency. Proc Natl Acad Sci U S A. 2019;116:1241–50. 30626647 10.1073/pnas.1816143116 PMC6347702

[41] Koga M. 1,5-Anhydroglucitol and glycated albumin in Glycemia. in 269–301 (2014). 10.1016/B978-0-12-800263-6.00007-0. 10.1016/b978-0-12-800263-6.00007-0 24938022

[42] Liu L, et al. Increased 1,5-Anhydroglucitol predicts glycemic remission in patients with newly diagnosed type 2 diabetes treated with short-term intensive insulin therapy. Diabetes Technol Ther. 2012;14:756–61. 22731793 10.1089/dia.2012.0055 PMC3429328

[43] Roberts DJ, Miyamoto S. Hexokinase II integrates energy metabolism and cellular protection: Akting on mitochondria and TORCing to autophagy. Cell Death Differ. 2015;22(2):248–57. 10.1038/cdd.2014.173. 25323588 10.1038/cdd.2014.173 PMC4291497

[44] Jeon JH, Hong CW, Kim EY, Lee JM. Current understanding on the metabolism of neutrophils. Immune Netw. 2020;20:1–13. 10.4110/in.2020.20.e46 PMC7779868 33425431

[45] Pajak B, et al. 2-deoxy-d-glucose and its analogs: from diagnostic to therapeutic agents. Int J Mol Sci. 2019;21:234. 31905745 10.3390/ijms21010234 PMC6982256

[46] Morava E, et al. Impaired glucose-1,6-biphosphate production due to bi-allelic PGM2L1 mutations is associated with a neurodevelopmental disorder. Am J Hum Genet. 2021;108:1151–60. 33979636 10.1016/j.ajhg.2021.04.017 PMC8206387

[47] Boulanger C, et al. Successful use of empagliflozin to treat neutropenia in two G6PC3-deficient children: impact of a mutation in SGLT5. J Inherit Metab Dis. 2022;45:759–68. 35506446 10.1002/jimd.12509 PMC9540799

[48] Veiga-da-Cunha M, Wortmann SB, Grünert SC, Van Schaftingen E. Treatment of the neutropenia associated with GSD1b and G6PC3 deficiency with SGLT2 inhibitors. Diagnostics. 2023;13(10):1803. 10.3390/diagnostics13101803 PMC10217388 37238286

[49] Moradian N, et al. Severe congenital neutropenia due to G6PC3 deficiency: early and delayed phenotype of a patient. Allergy Asthma Clin Immunol. 2023;19:1–8. 37296469 10.1186/s13223-023-00804-4 PMC10257254

[50] Notarangelo LD, et al. Severe congenital neutropenia due to G6PC3 deficiency: early and delayed phenotype in two patients with two novel mutations. Ital J Pediatr. 2014;40:1–6. 25391451 10.1186/s13052-014-0080-8 PMC4234865

[51] Yildirmak ZY, Ozcelik G, Ozagari AA, Genc DB, Onay H. Amyloidosis in a patient with congenital neutropenia because of G6PC3 deficiency. J Pediatr Hematol Oncol. 2022;44:E431–433. 34224517 10.1097/MPH.0000000000002237

[52] Glasser CL, et al. Phenotypic heterogeneity of neutropenia and gastrointestinal illness associated with G6PC3 founder mutation. J Pediatr Hematol Oncol. 2016;38:e243–7. 27571123 10.1097/MPH.0000000000000660

[53] Smith BN, et al. Phenotypic heterogeneity and evidence of a founder effect associated with G6PC3 mutations in patients with severe congenital neutropenia. Br J Haematol. 2012;158:146–9. 22469094 10.1111/j.1365-2141.2012.09110.x PMC4533883

[54] Banka S, Wynn R, Newman WG. Variability of bone marrow morphology in G6PC3 mutations: is there a genotype–phenotype correlation or age-dependent relationship? Am J Hematol. 2011;86:235–7. 21264919 10.1002/ajh.21930

[55] Lin SR, Pan CJ, Mansfield BC, Chou JY. Functional analysis of mutations in a severe congenital neutropenia syndrome caused by glucose-6-phosphatase-β deficiency. Mol Genet Metab. 2015;114:41–5. 25492228 10.1016/j.ymgme.2014.11.012 PMC4794745

[56] Pinho SS, Alves I, Gaifem J, Rabinovich GA. Immune regulatory networks coordinated by glycans and glycan-binding proteins in autoimmunity and infection. Cell Mol Immunol. 2023;20:1101–13. 37582971 10.1038/s41423-023-01074-1 PMC10541879

[57] Yu F, Tan WJ, Lu Y, MacAry PA, Loh KS. The other side of the coin: leveraging Epstein–Barr virus in research and therapy. Oral Oncol. 2016;60:112–7. 27531881 10.1016/j.oraloncology.2016.07.010 PMC7108324

[58] Alizadeh Z, et al. Two cases of syndromic neutropenia with a report of novel mutation in G6PC3. Iran J Allergy Asthma Immunol. 2011;10:227–30. 21891829

[59] Fernandez BA, et al. Adult siblings with homozygous G6PC3 mutations expand our understanding of the severe congenital neutropenia type 4 (SCN4) phenotype. BMC Med Genet. 2012;13:111. 23171239 10.1186/1471-2350-13-111 PMC3523052

[60] Lédeczi Z, Pittner R, Kriván G, Kardon T, Legeza B. Empagliflozin restores neutropenia and neutrophil dysfunction in a young patient with severe congenital neutropenia type 4. J Allergy Clin Immunol Pract. 2023;11:344–e3461. 36309187 10.1016/j.jaip.2022.10.019

[61] Hiwarkar P, et al. SLGT2 inhibitor rescues myelopoiesis in G6PC3 Deficiency. J Clin Immunol. 2022;42:1653–9. 35838821 10.1007/s10875-022-01323-4

[62] Dale DC, Bolyard AA, Makaryan V. The promise of novel treatments for severe chronic neutropenia. Expert Rev Hematol. 2023;16:1025–33. 37978893 10.1080/17474086.2023.2285987

