# Supplementary material for: Molecular and Clinical Characterization of a Founder Mutation Causing G6PC3 Deficiency
Source: J Clin Immunol. 2024 Dec 4;45(1):53. doi: 10.1007/s10875-024-01836-0 (PMC11618172; doi:10.1007/s10875-024-01836-0)
Supplement: Supplementary file 1 — (DOCX 2.39 MB) [file 10875_2024_1836_MOESM1_ESM.docx]

**Supplementary material**


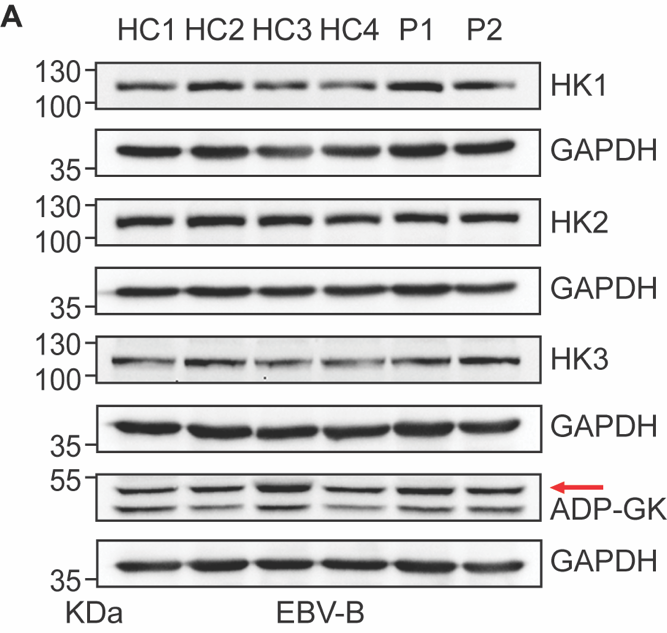


**Supplementary Figure 1:** Expression of low-K_M_ hexokinases and ADP-glucokinase in whole cell lysate of EBV-B cells derived from healthy controls (HC) and G6PC3 deficient patients (P) by western blot. GAPDH was used as the loading control.


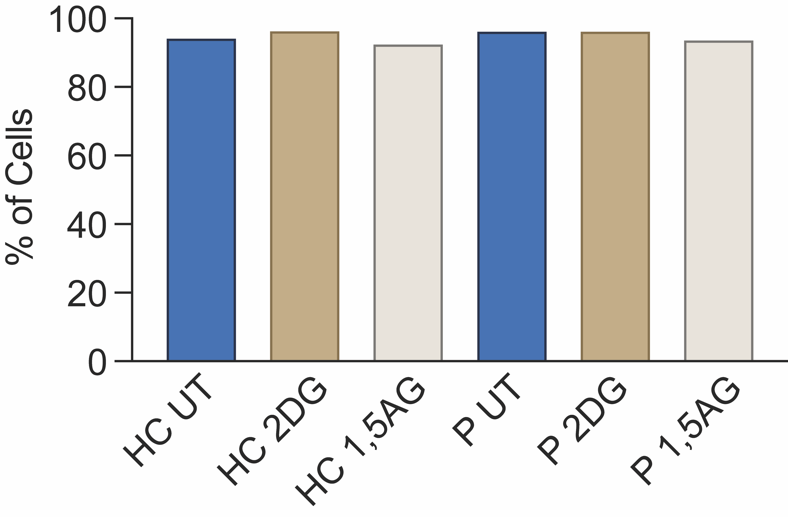


**Supplementary Figure 2:** EBV-B cells from one healthy control (HC) and one G6PC3 deficient patient (P) were treated with 2-DG or 1,5-AG for five days prior to the measurement of viability rate by flow cytometry. The singlet population was gated before quantification of the percentage of viable cells.
